# Tumor necrosis factor alpha inhibitors have no effect on a human T-lymphotropic virus type-I (HTLV-I)-infected cell line from patients with HTLV-I-associated myelopathy

**DOI:** 10.1186/s12865-017-0191-2

**Published:** 2017-02-03

**Authors:** Shoichi Fukui, Hideki Nakamura, Yoshiko Takahashi, Naoki Iwamoto, Hiroo Hasegawa, Katsunori Yanagihara, Tatsufumi Nakamura, Akihiko Okayama, Atsushi Kawakami

**Affiliations:** 1 Department of Immunology and Rheumatology, Nagasaki University Graduate School of Biomedical Sciences, Nagasaki, Japan; 2 Department of Laboratory Medicine, Nagasaki University Graduate School of Biomedical Sciences, Nagasaki, Japan; 3 Department of Human Community, Faculty of Social Welfare, Nagasaki International University, Sasebo, Japan; 4 Department of Rheumatology, Infectious Diseases and Laboratory Medicine, Faculty of Medicine, University of Miyazaki, Miyazaki, Japan

**Keywords:** HTLV-I, TNF-α inhibitor, Cytokine, Chemokine, Proviral load

## Abstract

**Background:**

While tumor necrosis factor alpha (TNF-α) inhibitors (TNFi) and other biologics are very effective against autoimmune diseases, they can also cause infectious diseases. Therefore, it is important to clarify whether the TNFi sometimes used to treat patients with rheumatoid arthritis (RA) complicated with human T-lymphotropic virus type-I (HTLV-I) infection have the unintended side effect of promoting HTLV-I proliferation.

**Methods:**

We used the HTLV-I-infected cell line HCT-5, derived from spinal fluid cells of a patient with HTLV-I associated myelopathy, to evaluate the production of cytokines and chemokines, TNF-α receptor (TNFR), the expression of HTLV-I associated genes, the HTLV-I proviral load (PVL), the expression of HTLV-I structural protein, and apoptosis. We used Jurkat cells as a control.

**Results:**

Supernatants of HCT-5 showed time-dependent elevations of IL-6, RANTES and ICAM-1. HCT-5 supernatants treated with infliximab, adalimumab, etanercept (ETN), golimumab and certolizumab pegol showed no significant differences in the levels of these molecules compared to the control. Neither TNFR1 nor TNFR2 expression was altered by any TNFi treatment, relative to phosphate-buffered saline (PBS) treatment, with the exception that TNFR2 was significantly decreased and internalized in HCT-5 cells by ETN treatment. The HTLV-I associated genes Tax and HBZ and the PVL levels were not significantly changed. Immunofluorescence staining of HCT-5 for an HTLV-I-associated protein, GAG, was also not significantly different between any of the TNFi treatments and the PBS treatment. DNA ladders as an index of apoptosis were not detected. Apoptotic cells were not increased by the addition of any TNFi.

**Conclusions:**

In vitro, TNFi did not affect the cytokine profiles, expression of associated genes and proteins, proviral load or apoptosis of HCT-5 cells. The results suggested that TNFi treatment of RA patients complicated with HTLV-I might have no effect on HTLV-I infection.

## Background

Human T-lymphotropic virus type-I (HTLV-I) is a retrovirus that infects 10 to 20 million people worldwide [1]. There are areas in sub-Saharan Africa, the Caribbean, and South America where >1% of the general population is infected, [2] and southwestern Japan including Nagasaki Prefecture is one of the endemic areas [3]. Although the majority of infected people remain asymptomatic, HTLV-I is associated with severe diseases such as adult T-cell leukemia/lymphoma (ATL) and HTLV-I-associated myelopathy (HAM). Many strategies have been evaluated for the treatment of ATL and HAM, but no treatments have shown sufficient efficacy.

Tumor necrosis factor alpha (TNF-α) inhibitors (TNFi) are an important agent for a number of inflammatory conditions, including rheumatoid arthritis (RA), [4] ankylosing spondylitis, [5] and inflammatory bowel disease [6]. However, multiple adverse effects of TNF-α inhibition have been identified, including infections, malignancies, and the induction of autoimmunity and demyelinating diseases. With respect to viral infection, hepatitis B virus (HBV) occasionally reactivates, and a flare of HBV disease may occur [7].

However, it is unknown whether HTLV-I proliferates and whether HTLV-I-associated diseases worsen when biologics including TNFi are used. Answers to these questions are needed by clinicians who use biologics. In Japan, approximately one million individuals are carriers of HTLV-I, [8] which means that one person per 100 individuals has an HTLV-I infection. In an RA cohort study, 21.3% of the RA patients were treated with a TNFi [9]. Whenever possible, clinicians would prefer to avoid the use of TNFi to treat HTLV-I-infected patients, but in the case of patients with RA complicated by HTLV-I infection, the use of TNFi is unavoidable due to the high prevalence of both conditions. Because the use of biologics for such patients is relatively new, the problem of biologics–induced enhancement of HTLV-I in RA patients is also a fairly new concern. In addition, a significant increase in the standardized incidence ratio for malignant lymphoma was identified in a Japanese nationwide cohort of patients treated with biological disease-modifying anti-rheumatic drugs (DMARDs) including TNFi, [10] but that study did not reveal whether the standardized incidence ratio for ATL increased. We searched for cases of ATL or HAM patients treated with a TNFi as an autoimmune disease treatment by conducting a PubMed search, but to the best of our knowledge, there were no such reports with the exception of one smoldering ATL case [11].

For the above reasons, it is necessary establish whether a TNFi can be used safely to treat patients with inflammatory diseases such as RA complicated with HTLV-I infection. For this purpose, we plan to perform both in vitro and clinical investigations to ascertain the safety of TNFi treatment in patients with HTLV-I infection. To this end, we herein assessed changes in the cytokine profiles, associated proteins, proviral load (PVL), and apoptosis in an HTLV-I infected cell line treated with several different TNFi.

## Methods

### Cell lines

The HTLV-I-infected T-cell line HCT-5 derived from the cerebrospinal fluid cells of a HAM patient was used. This cell line is interleukin (IL)-2-dependent and was maintained in RPMI 1640 (Wako Pure Chemical Industries, Tokyo) containing 20% fetal bovine serum (FBS) (Thermo Fisher Scientific, Waltham, MA) and 1% penicillin/streptomycin (Thermo Fisher Scientific) supplemented with 100 U/ml of recombinant human IL-2 (kindly provided by Shionogi & Co., Osaka, Japan) and L-glutamine (Sigma-Aldrich, St. Louis, MO). Jurkat cells, a human T-cell lymphoblast-like cell line, were used as a control cell line. THP-1, a human monocytic leukemia cell line, was used to ascertain whether the TNFi worked as expected to inhibit TNF-α.

These cell lines were maintained in RPMI 1640 containing 10% FBS and 1% penicillin/streptomycin. All cell lines were incubated in a humidified incubator at 37 °C with an atmosphere of 5% carbon dioxide.

### Reagents and TNFi

We purchased infliximab (IFX; Centocor, Malvern, PA), adalimumab (ADA; Abbott, Abbott Park, IL), etanercept (ETN; Amgen, Thousand Oaks, CA), golimumab (GLM; Janssen Biotech, Horsham, PA) and certolizumab pegol (CZP; UCB Pharma, Brussels, Belgium). We used lipopolysaccharide (LPS; Sigma-Aldrich) to stimulate the THP-1 cells.

### Multiplex cytokine/chemokine bead assays

We performed multiplex cytokine/chemokine bead assays using culture supernatants and Milliplex MAP Human Cytokine/Chemokine Panel 1 Pre-mixed 41Plex (Merck Millipore, Darmstadt, Germany). We analyzed the results with a Bio-Plex® MAGPIX™ Multiplex Reader (Bio-Rad, Hercules, CA) according to the manufacturer’s instructions. The cytokines/chemokines that could be measured by the Milliplex MAP Human Cytokine/Chemokine Panel 1 Pre-mixed 41Plex include vascular endothelial growth factor (VEGF), tumor necrosis factor-beta (TNF-β), TNF-α, transforming growth factor-α (TGF-α), regulated on activation, normal T cell expressed and secreted (RANTES), platelet-derived growth factor (PDGF)-AB/BB, PDGF-AA, macrophage inflammatory protein (MIP)-1α, macrophage inflammatory protein (MIP)-1β, macrophage-derived chemokine (MDC) (C-C motif chemokine [CCL]22), monocyte chemotactic protein-3 (MCP-3), monocyte chemotactic protein-1 (MCP-1), inducible protein-10 (IP-10), IL-2, -3, -4, -5, -6, -7, -8. -9, -10, -12, -13, -15, -17, IL-12 (p70), IL-12 (p40), IL-1ra, IL-1β, IL-1α, interferon-gamma (IFN-γ), interferon-α2 (IFN-α2), growth-related oncogene (GRO), granulocyte macrophage colony stimulating factor (GM-CSF), granulocyte colony stimulating factor (G-CSF), fractalkine, Fms-related tyrosine kinase 3 ligand (Flt-3 L), fibroblast growth factor (FGF)-2, eotaxin, epidermal growth factor (EGF), and soluble CD40L (sCD40L). We used the Bio-Plex Pro Human Cytokine Group II x-Plex Panel (Bio-Rad) to determine the serum levels of intercellular adhesion molecule (ICAM)-1, vascular cell adhesion molecule (VCAM)-1, IL-18, and stromal cell-derived factor (SDF)-1α.

### Cytokine enzyme-linked immunosorbent assays (ELISAs)

We used a cytokine ELISA immune assay to measure the levels of IL-6, TNF-α, sICAM-1/CD54 and CCR5/RANTES in culture supernatants of HCT-5 and Jurkat cells to which we had added five types of TNFi. The IL-6, TNF-α, sICAM-1/CD54 and CCR5/RANTES Quantikine ELISA kits were purchased from R&D Systems (Minneapolis, MN). The level of each cytokine was determined according to the manufacturer’s instructions, and then the optical density at 450 nm was measured.

### TNF-α receptor analysis

We performed fluorescence-activated cell sorting (FACS) analysis to examine the cell surface expression of TNF-R1 and -R2, using fluorescein isothiocyanate (FITC)-conjugated anti-CD120a (TNF-R1) and anti-CD120b (TNF-R2) human monoclonal antibodies (mAbs; MBL International, Woburn, MA). FITC-conjugated mouse IgG1 was used as an isotype control (BD Biosciences, San Jose, CA). Experiments were performed using a FACS Canto II Flow Cytometer and FACS Diva software (BD Biosciences).

### RNA extraction and quantitative reverse transcription PCR for gene expression

We extracted total RNA from HCT-5 and Jurkat cells by using a Kingfisher Pure RNA Blood kit (Thermo Fisher Scientific) according to the manufacturer’s instructions, and the total RNA was applied for cDNA synthesis with a SuperScript VILO cDNA Synthesis Kit (Invitrogen, Carlsbad, CA). Reverse transcription was performed with 1 μg of total RNA at 25 °C for 10 min, 42 °C for 60 min, and 85 °C for 5 min, in a final volume of 20 μl.

The expressions of viral Tax (forward, 5′-CCCACTTCCCAGGGTTTGGA-3′; reverse, 5′-GGCCAGTAGGGCGTGA-3′; probe, 5′-FAM-CCAGTCTACGTGTTTGGAGACTGTGTACA-TAMRA-3′), and HTLV-I bZIP factor (HBZ) mRNAs (forward, 5′-CTCAGGGCTGTTTCGATGCT-3′; reverse, 5′-GCCCGTCCACCAATTCCT-3′; probe, 5′-FAM-CCTGTGTCATGCCCGGAGGACC-TAMRA-3′), and porphobilinogen deaminase (PBGD, forward, 5′-AACCAGCTCCCTGCGAAGA-3′; reverse, 5′-CCAGGATGATGGCACTGAACT-3′; probe 5′-FAM-ACTCCTGAACTCCAGATGCGGGAACT-TAMRA-3′) were measured using a cDNA template with LightCycler 480 probes Master Mix (Roche Diagnostics, Mannheim, Germany) and a LightCycler480 PCR System (Roche Diagnostics).

After 50 cycles, the absolute amounts of Tax, HBZ, and PBGD mRNA were interpolated from standard curves generated by the dilution method using plasmids derived from a clone transfected with a pCR2.1-TOPO Vector (Life Technologies, Tokyo, Japan) containing amplicons from the Tax, HBZ, and PBGD genes, respectively. To normalize the results for variability in the concentration and integrity of the RNA and cDNA, we used the PBGD gene as an internal control for each sample.

### HTLV-I proviral load (PVL)

We extracted the genomic DNA of cells using Qiagen DNA Blood Mini kits (Qiagen, Crawley, UK). The quantitative polymerase chain reaction (qPCR) detection for HTLV-I was performed as described previously [12–15]. Briefly, primers were set in the pX region, and the density of the template was 30 ng per reaction. PVL was quantified using the Tax primer and probe. The PVL was normalized using β-globin and is presented as a percentage.

### Immunofluorescence

The HCT-5 cells were incubated for 10 min in phosphate-buffered saline (PBS) containing 4% paraformaldehyde at 4 °C and immersed in methanol at −20 °C for 10 min. After blocking in 5% normal horse serum in PBS, the cells were incubated in the diluted primary antibodies for 1 h at room temperature, followed by incubation in FITC-labeled and tetramethylrhodamine isothiocyanate (TRITC)-labeled secondary antibodies supplemented with Hoechst dye 33258 for nuclear staining. After being washed in PBS, the cells were mounted in Vectashield mounting medium (Vector Laboratories, Burlingame, CA) and scanned using a BIOREVO BZ-9000 fluorescence microscope (Keyence, Tokyo, Japan).

The following antibodies were used as primary antibodies: TNF Receptor 1 Polyclonal Antibody (Bioss Antibodies, Woburn, MA), TNFR2 Polyclonal Antibody (Proteintech, Chicago, IL), and Monoclonal Mouse anti-HTLV-I p19, HTLV-I p28 antibody (Chemicon, Hofheim, Germany).

### Assessment of apoptosis

Apoptotic DNA ladders were detected by using an Apoptotic DNA Ladder Kit (Sigma-Aldrich). Briefly, the cultured HCT-5 cells in six-well plates were lysed and mixed with isopropanol. After the plates were washed with PBS, the pre-warmed elution buffer was applied to obtain DNA. For the DNA ladder detection, the samples were applied to a 1% agarose gel with ethidium bromide in 1× Tris, boric acid and EDTA-buffer. Apoptotic U937 cells in the kit were used as a positive control.

### Annexin V staining

The evaluation of apoptosis and cell death was performed by staining with propidium iodide (PI) (MBL, Nagoya, Japan) and phycoerythrin (PE)-conjugated annexin V (MBL). After being washed with PBS, the cells were stained by PI and PE-conjugated annexin V for 15 min in ambient air. A FACS analysis was performed using a FlowSight Imaging Flow Cytometer (Merck-Millipore, Germany). PI-negative and annexin V-positive cells were defined as apoptotic cells. We calculated the percentage of apoptotic cells among all cells.

### Statistical analysis

We used a Student’s *t*-test to determine the significance of differences in the levels of IL-6, TNF-α, sICAM-1/CD54, CCR5/RANTES, Tax, HBZ, and PVL, and the percentage of apoptotic cells. *P*-values <0.05 were considered significant. Statistical analyses were performed with JMP Statistical Software, ver. 11 (SAS Institute, Cary, NC).

## Results

### Multiplex cytokine/chemokine bead assays

It was previously reported that HTLV-I-infected CD4+ T cells spontaneously secreted proinflammatory cytokines such as TNF-α and IFN-γ [16]. In the present study, to identify the types of cytokines and chemokines secreted by HCT-5 cells into the culture supernatant compared to those secreted by the Jurkat cells, we performed multiplex cytokine/chemokine bead assays. Because we previously ascertained that HCT-5 cells could be kept without stimulation by IL-2 for at least 96 h, [17] we used culture supernatants at 0, 24, 48, 72 and 96 h without IL-2 stimulation.

**Fig. 1:**
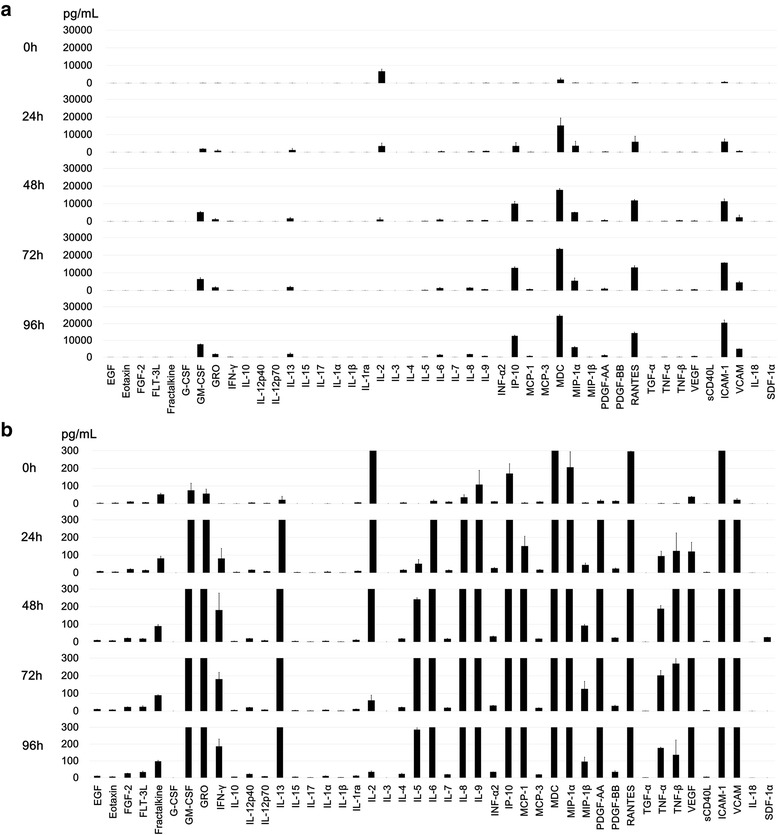
Cytokines and chemokines secreted by HCT-5 into the culture supernatant at 0, 24, 48, 72 and 96 h without any stimulation. **a** High-scale magnification and **b** low-scale magnification from three independent experiments. Error bars represent the standard deviations

In the HCT-5 cells, we observed time-dependent increases in the levels of IL-6, IP-10, MDC, MIP-1α, RANTES, ICAM-1, VCAM-1, TNF-α, and IFN-γ in the culture supernatants without any stimulation (Fig. 1a, b). In the Jurkat cells, there were no increases in these cytokines or chemokines, and most of the cytokines and chemokines were below measurable limits (data not shown).

### Changes in the cell count and cytokine and chemokine levels in the HCT-5 cells treated with each TNFi

**Fig. 2:**
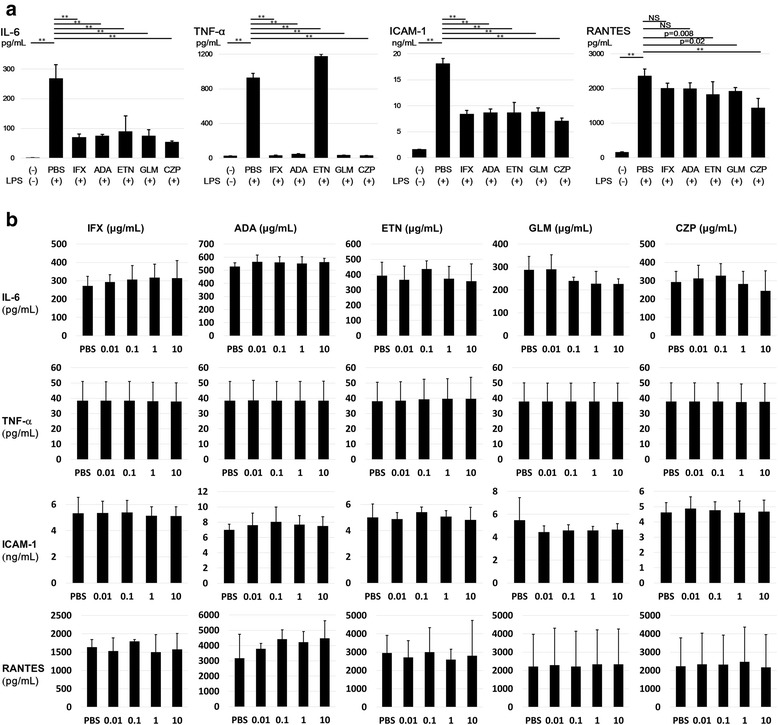
**a** THP-1 was stimulated by LPS (1 μg/mL) with each TNFi (10 μg/mL) and PBS to ascertain the effect of each TNFi. Forty-eight hours after stimulation, the levels of IL-6, TNF-α, ICAM-1 and RANTES were measured in the culture supernatants (three independent experiments). **p < 0.01, Student’s *t*-test. **b** The levels of IL-6, TNF-α, ICAM-1 and RANTES in the culture supernatants of HCT-5 without LPS stimulation were measured at 48 h after the addition of each TNFi (10 μg/mL) or PBS (three independent experiments). Error bars represent the standard deviations

Before using each TNFi on the HCT-5 cells, we ascertained whether it properly inhibited TNF-α in THP-1 cells. Forty-eight hours after the THP-1 cells were stimulated by LPS (1 μg/mL) with each TNFi, we measured the levels of IL-6, TNF-α, ICAM-1 and RANTES in the culture supernatants by ELISAs. TNF-α was inhibited by all of the TNFi except ETN. IL-6 and ICAM-1 in the culture supernatants were inhibited by all of the TNFi. There were significant decreases in the levels of RANTES in the ETN-, GLM- and CZP-treated cells (Fig. 2a).

To identify whether TNFi induced an increase or decrease in the total number of cells, we counted the cells under a light microscope. The cultured HCT-5 cells (1.0 × 10^6^) in six-well plates with each TNFi (10 μg/mL) and PBS increased time-dependently to approximately 1.5 × 10^6^ at 96 h. The HCT-5 cell line treated with each TNFi showed a time-dependent increase in cell counts at 0, 48 and 96 h, and there were no significant differences in the increases compared to those administered PBS alone (data not shown).

To determine the changes in the cytokines and chemokines in culture supernatants, we used ELISAs to measure IL-6, TNF-α, ICAM-1 and RANTES in the culture supernatants of HCT-5 and Jurkat cells 48 h after the addition of each TNFi or PBS. There were no significant differences in the levels of IL-6, TNF-α, ICAM-1 or RANTES in the culture supernatants of HCT-5 cells compared to those treated with PBS alone (Fig. 2b). In the Jurkat cells, there were also no differences (data not shown).

### Expressions of TNFR1 and -2 on HCT-5 and Jurkat cells treated with each TNFi

**Fig. 3:**
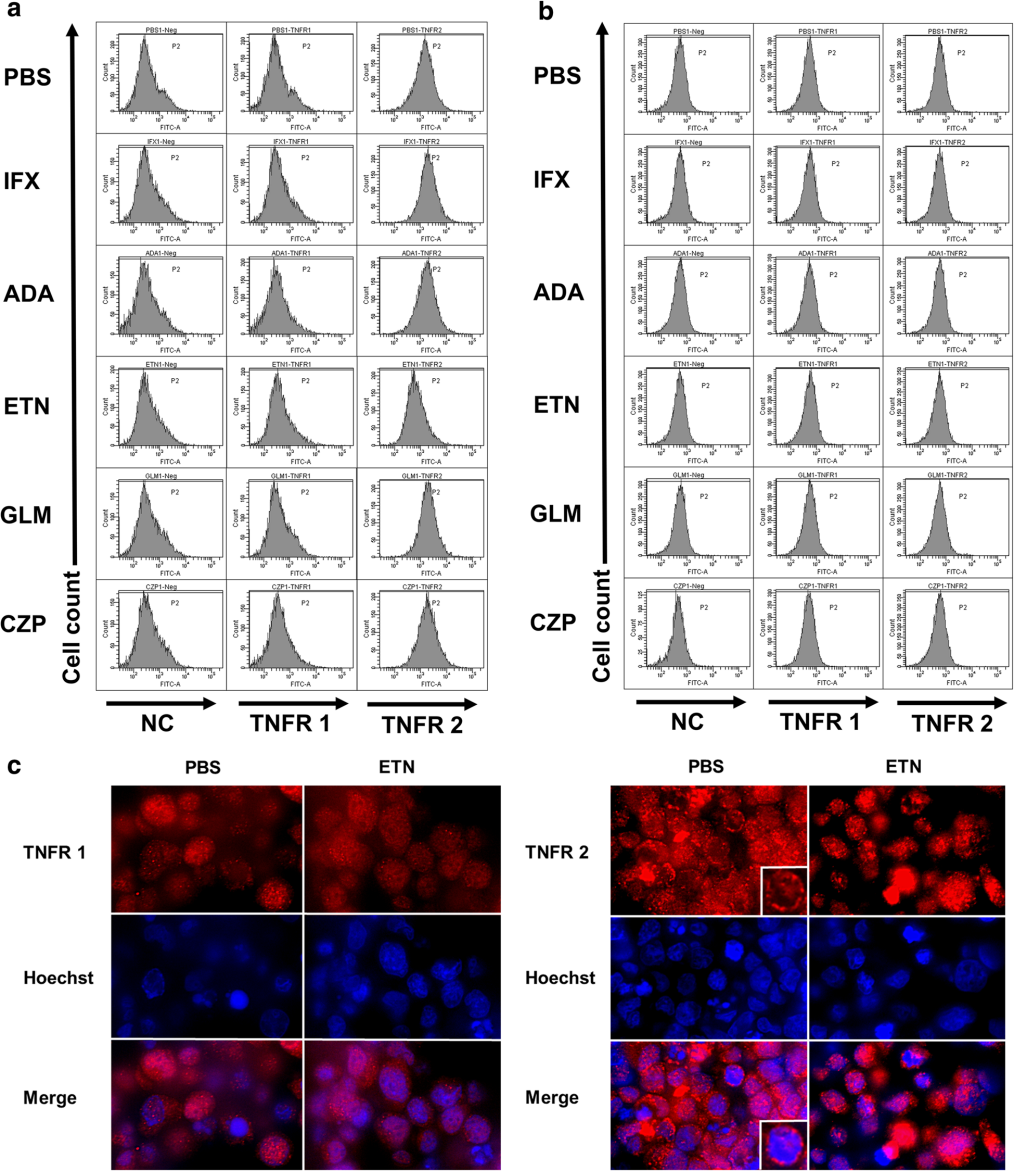
Expressions of TNFR1 and TNFR2 of HCT-5 (**a**) and Jurkat (**b**) cells by FACS analysis. **c** Immunofluorescence staining of TNFR1 and TNFR2 48 h after the addition of ETN (10 μg/mL) and PBS. Inset: A representative cell that was treated with PBS (three independent experiments)

To identify the changes in the expressions of TNF receptor (TNFR)1 and -2 caused by each TNFi, 48 h after the addition of each TNFi or PBS, we assessed the expressions of TNFR1 and -2 on HCT-5 cells by performing a FACS analysis (Fig. 3a). There were no significant differences in the expressions of TNFR1 for any of the TNFi compared to that with PBS. There were no significant differences in the expressions of TNFR2 except in the ETN-treated cells; in these cells, the TNFR2 expression was decreased (Fig. 3a). There were no significant differences in the expressions of TNFR1 or -2 in the Jurkat cells (Fig. 3b).

To assess the expressions of TNFR1 and -2 on the cell membrane and cytoplasm in HCT-5 cells treated with ETN, we performed immunofluorescence staining. In the HCT-5 cells treated with either ETN or PBS, TNFR1 was expressed in the cytoplasm and cell membrane. TNFR2 was expressed on the cell membrane of the PBS-treated cells but not on that of the ETN-treated cells. In the ETN-treated cells, TNFR2 was expressed mainly in the cytoplasm (Fig. 3c).

### No changes in the mRNAs of Tax or HBZ, or the PVL in HCT-5 cells treated with TNFi

**Fig. 4:**
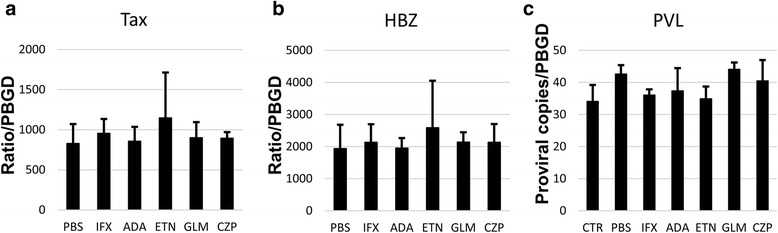
mRNA expression of Tax (**a**), HBZ (**b**) and PVL (**c**) 48 h after the addition of each TNFi (10 μg/mL) and PBS. (three independent experiments). PBGD: housekeeping gene. Error bars represent the standard deviations

The HTLV-I viral gene Tax is the gene for a potent transactivating protein that is required for the expression of the viral gene [18]. Another HTLV-I antisense-encoded gene, HBZ, along with its protein form, is also important for the proliferation of HTLV-I [19]. Therefore, to identify the TNFi-induced changes of Tax and HBZ in the HCT-5 and Jurkat cell lines, we measured the expressions of the mRNAs of Tax and HBZ at 48 h after the addition of each TNFi or PBS. There were no significant differences in the expressions of the mRNAs of Tax (Fig. 4a) or HBZ (Fig. 4b).

The PVL is the most frequently used marker for prognosis and disease progression in infected patients [2]. For example, the PVL is correlated with the progression of motor disability in HAM patients [20] Using the same method as used for Tax and HBZ, we measured the PVL at 48 h after the addition of each TNFi or PBS. None of the TNFi increased the PVL significantly compared with PBS, although the PVL of the HCT-5 cells without TNFi was 42.7 proviral copies per cell (Fig. 4c). In the Jurkat cells, provirus was not detected.

### No effects on GAG expression by any of the TNFi in HCT-5 cells

**Fig. 5:**
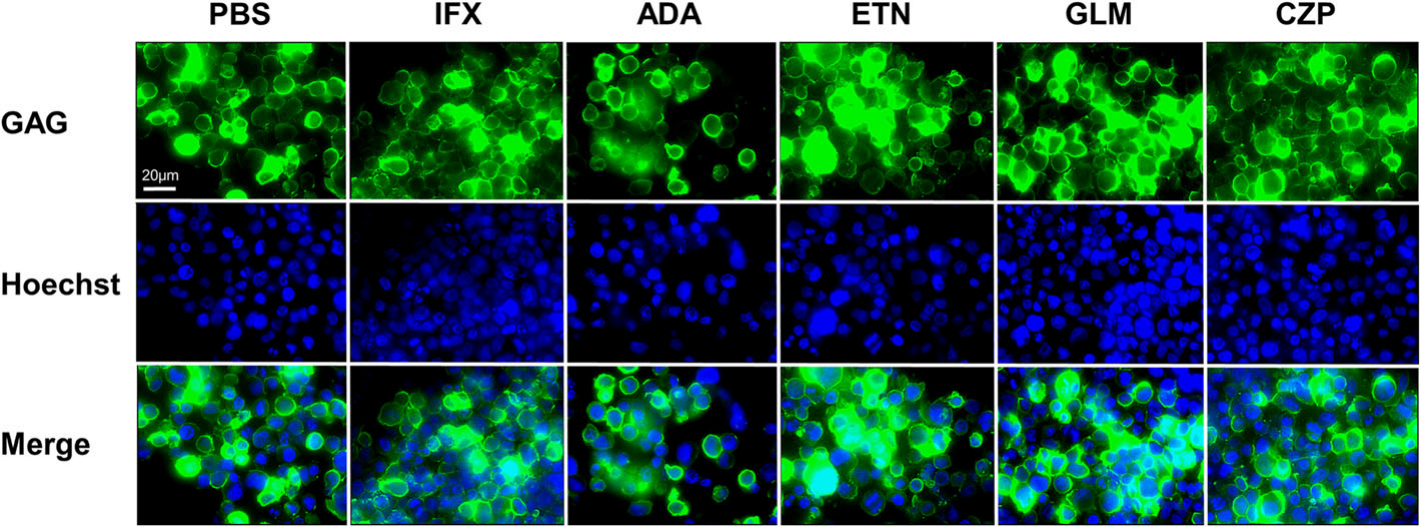
After HCT-5 cells were stimulated with PBS or 10 μg g/ml of a TNFi for 48 h, they were treated with a mouse monoclonal anti-HTLV-I p19, p28 (GAG) antibody followed by donkey anti-mouse FITC-labeled secondary antibody. Hoechst 33258 was used for counterstaining of the nucleus (merged view). Bar: 20 μm (three independent experiments)

The HTLV-I structural protein GAG is processed into the virion core proteins [19]. To assess the potential effects of the TNFi on GAG expression, we performed HTLV-I p19 and p28 (GAG) staining 48 h after the addition of each TNFi (10 μg/ml) or PBS. No significant differences in GAG expression were observed (Fig. 5).

### Assessment of apoptosis in HCT-5 cells

**Fig. 6:**
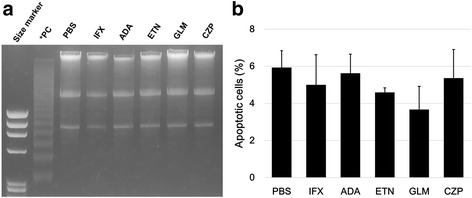
**a** After HCT-5 cells were stimulated with PBS or 10 μg/ml of a TNFi for 48 h, the cells were subjected to an apoptotic ladder experiment. PC: positive control. U937 cells were treated with camptothecin. **b** After HCT-5 cells were stimulated with PBS or 10 μg/ml of a TNFi for 48 h, we evaluated the percentage of apoptotic HCT-5 cells defined as negative propidium iodide and positive phycoerythrin-conjugated annexin V. Error bars represent the standard deviations

To assess the apoptosis in HCT-5 cells treated with each TNFi or PBS, we performed apoptotic DNA ladder detection. Apoptotic DNA ladders were not detected 48 h after the addition of any of the TNFi (10 μg/ml) or PBS (Fig. 6a). In addition, we performed annexin V staining of HCT-5 48 h after the addition of each of the TNFi (10 μg/ml) or PBS (Fig. 6b). Approximately 5% of the cells were apoptotic in each case, with no significant differences in the percentage of apoptotic cells among the TNFi and PBS treatments.

## Discussion

Our present findings demonstrated that none of the TNFi induced any significant change in the proliferation of HTLV-I-infected cells, the cytokine or chemokine production, the expression of TNFR1, the expressions of Tax or HBZ, the PVL, the level of GAG, or apoptosis, compared to the values in the HTLV-I-infected cells treated with PBS alone.

When a TNFi is necessary to treat RA patients complicated with HTLV-I infection, the occurrence and exacerbation of both ATL and HAM are a concern. Clinically, two RA cases complicated with HTLV-I infection were reported to show no increase in PVL by TNFi treatment [21] In another report, a patient with smoldering ATL and HTLV-I-associated arthropathy that was refractory to corticosteroid, DMARDs and rituximab therapy was treated successfully with ETN [11] These reports are valuable in that they show the safety of TNFi for HTLV-I infections, but at the same time their value is limited because they are case reports rather than studies on large numbers of patients.

The risk of TNFi-induced ATL must be considered. TNF-α was originally recognized in 1975 for its ability to lyse tumors in a variety of in vitro and mouse models, [22] and TNFi were thus assumed to induce tumors. In contrast, a paradoxical tumor-promoting role of TNF has also become apparent [23]. The tumorigenesis of TNFi is therefore more complex than original thought.

Tax is thought to induce ATL by promoting cellular proliferation through the enhancement of cellular survival and impairment of DNA damage-repair mechanisms [24]. In the process of viral proliferation, HTLV-I regulates not only Tax [25] but also HBZ [26]. Our present results demonstrated that the levels of HBZ in HCT-5 cells were not significantly changed by treatment with various TNFi. Because Tax and HBZ were not increased following treatment with any of the TNFi in this study, TNFi may not induce ATL. In addition, PVL was not increased by the treatments in this study. Since there was no TNFi-induced increase in either Tax or HBZ, it seems reasonable that there was also no increase in PVL or GAG.

The inhibition of apoptosis is important for viral proliferation, and a functional inactivation of p53 is induced by Tax [27]. In regard to the relationship between apoptosis and TNFi, it has been reported that the apoptosis of T lymphocytes was induced by IFX [28] and ADA [29]. in a human-mouse chimeric model. In addition, IFX activates the p53 gene, whereas ETN does not [30]. In the present study, none of the TNFi appeared to enhance apoptosis, although we hypothesize that all TNFi except for ETN are capable of inducing the apoptosis of T lymphocytes, including HCT-5 cells, and inhibiting the occurrence of ATL.

Finally, we must consider the risk of HAM due to TNFi treatment. Several reports have examined the relation between inflammation and HTLV-I infection. Tax boosts the expression of the Th1 master regulator T box transcription factor and consequently promotes the production of IFN-γ [31] HBZ has been shown to induce systemic inflammation by impairing the suppressive function of Treg cells via an interaction with Foxp3 and NFAT in HBZ-transgenic mice [32] Clinically, patients with HAM have high circulating levels of TNFα- and IL-2-secreting HTLV-I-specific CD4+ T cells [16] In the present study, we observed the elevated production of multiple cytokines and chemokines, including IL-6, RANTES, ICAM-1, TNF-α and IFN-γ, in the supernatant of HCT-5 cells.

In this vein, it should be noted that immunosuppressive and immunomodulatory therapies have been reported to have beneficial effects in patients with HAM, [33] suggesting that the production of cytokines and chemokines is important in the pathophysiology of this disorder. Although we did not observe a reduction of cytokines or chemokines induced by any of the TNFi in the present study, on the basis of previous studies it appears that TNFi could have the effect of alleviating HAM.

With respect to our results showing that TNFR2 was decreased and internalized in the HCT-5 cells treated with ETN, it is apparent that among the TNFi, ETN had a different effect on TNFR2. ETN is a soluble p75 TNF receptor fusion protein, [34] while the other TNFi are monoclonal antibodies directed against TNF-α [35]. This difference may have affected the decreased expression of TNFR2 in the ETN-treated HCT-5 cells in the present study through a mechanism described by R.E. Kast in 2005 [36].

This study has some limitations. Because this was an in vitro study using a cell line established from a HAM patient, the results do not necessarily reflect the clinical effects of TNFi in vivo. In addition, our findings have no validity in ATL cell lines because there are some etiological differences between HAM and ATL. For example, the expression of Tax is frequently disrupted in ATL, [37] but HBZ is uniformly expressed in ATL [38]. The questions of whether TNFi directly induce ATL and whether TNFi treatment presents a risk for the development of ATL in daily practice remain to be addressed. Tax has the ability to cause ATL through the induction of cell-cycle progression, DNA damage, impairment of DNA repair, and cellular transformation [39]. The present study did not assess these factors. However, our findings showed no increase of Tax, and we thus speculate that Tax-mediated effects causing ATL did not increase. In this regard, we have begun clinical investigations in HTLV-I infected RA patients treated with biologics including TNFi.

## Conclusions

We have shown that TNFi did not affect the cytokine profiles, the mRNA of HTLV-I-associated proteins, the PVL or the apoptosis of HCT-5 cells. This study is the first in vitro investigation to evaluate the effects of TNFi on HTLV-I-infected cells. Further in vitro and in vivo studies are needed to assess the effects of TNFi on HTLV-I to clinically ascertain the safety of TNFi treatments in RA patients complicated with HTLV-I infection.

## Abbreviations

ADA: Adalimumab
CZP: Certolizumab pegol
ETN: Etanercept
GLM: Golimumab
HAM: HTLV-I associated myelopathy
HTLV-I: Human T-lymphotropic virus type-I
IFX: Infliximab
PVL: Proviral load
TNFi: Tumor necrosis factor alpha inhibitor
TNFR: Tumor necrosis factor alpha receptor
